# Knockdown of LINC01138 protects human chondrocytes against IL‐1β‐induced damage by regulating the hsa‐miR‐1207‐5p/KIAA0101 axis

**DOI:** 10.1002/iid3.744

**Published:** 2022-12-15

**Authors:** Jiangtao Zhang, Genbing Lv

**Affiliations:** ^1^ Three Departments of Knee Injury, Luoyang Orthopedic Hospital of Henan Province Orthopedic Hospital of Henan Province Luoyang Henan P. R. China; ^2^ Department of Orthopedics, Sun Si Miao Hospital of Beijing University of Chinese Medicine Tongchuan Traditional Chinese Medicine Hospital Tongchuan Shanxi P. R. China

**Keywords:** hsa‐miR‐1207‐5p, IL‐1β, KIAA0101, LINC01138, osteoarthritis

## Abstract

**Introduction:**

Long intergenic non‐protein coding RNA 1138 (LINC01138) plays a vital role in human cancers. In this study, we aimed to investigate the effect of LINC01138 on the progression of osteoarthritis (OA) and explore its potential mechanism of action.

**Methods:**

The expression of LINC01138, hsa‐miR‐1207‐5p, and KIAA0101 in OA tissues and normal tissues was analyzed using GSEA datasets and confirmed in human specimens. Human chondrocytes were treated with interleukin (IL)‐1β to establish an OA cell model. Quantitative real time PCR(qRT‐PCR), enzyme‐linked immunosorbent assay, and western blotting analyses were performed to evaluate the role of LINC01138, hsa‐miR‐1207‐5p, and KIAA0101 during extracellular matrix (ECM) protein degeneration and cellular inflammatory response. The target relationship was predicted using DIANA‐TarBase and TargetScan. The binding effects were verified by dual‐luciferase reporter assay.

**Results:**

LINC01138 expression was higher in OA tissues than in normal controls. LINC01138 levels increased in chondrocytes treated with IL‐1β. Silencing of LINC01138 attenuated the IL‐1β‐induced decrease in Col2α1, aggrecan, and sulphated glycosaminoglycan (sGAG), and inhibited the IL‐1β‐induced increase in matrix metalloproteinase (MMP)‐13, IL‐6, and tumor necrosis factor (TNF)‐α. miR‐1207‐5p is weakly expressed in OA tissues and cell models. The inhibition of hsa‐miR‐1207‐5p, a target of LINC01138, attenuated the effects of LINC01138 silencing on chondrocyte ECM degeneration and inflammatory responses. Silencing KIAA0101, a target of hsa‐miR‐1207‐5p, alleviated the effect of hsa‐miR‐1207‐5p on chondrocyte ECM degeneration and inflammatory responses. Furthermore, silencing of KIAA0101 inhibited the JAK/STAT and Wnt signaling pathways.

**Conclusion:**

Silencing LINC01138 protected chondrocytes from IL‐1β‐induced damage, possibly by regulating the hsa‐miR‐1207‐5p/KIAA0101 axis.

## Graphical abstract

Silence of LINC01138 protected chondrocytes from IL‐1β‐induced damage possibly by regulating the hsa‐miR‐1207‐5p/KIAA0101 axis.

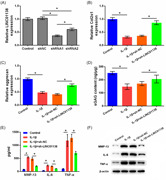

## 1 INTRODUCTION

Osteoarthritis (OA) is a degenerative joint disease that seriously endangers the life, work, and quality of life of middle‐aged and elderly individuals. According to a previous report, more than 250 million people worldwide have OA.^1^ The main clinical manifestations of this disease are joint pain, stiffness, swelling, deformity, and dysfunction. Symptomatic relief remains the main goal of disease management.^2^, ^3^ Chondrocytes are an important cell type in cartilage and their dysfunction is related to the occurrence of OA.^4^ OA has been reported to be the result of abnormal apoptosis of chondrocytes caused by an imbalance in the degradation and synthesis coupling of chondrocytes. In addition, the extracellular matrix (ECM) and chondrocytes are involved in the interaction between biological factors and mechanical injuries.^5^ However, the aetiopathogenesis of OA has not yet been completely elucidated. Consequently, investigating the pathological mechanism of action of OA is essential for identifying new strategies for early diagnosis and treatment.

Long noncoding RNAs (lncRNAs) are evolutionarily conserved RNAs that belong to the family of ncRNAs and are characterized by a lack of functional protein‐coding ability.^6^ It has been reported that lncRNAs are aberrantly expressed in OA and play an important role in OA progression.^7^, ^8^ Long intergenic non‐protein coding RNA 1138 (LINC01138), located at 1q21.2, is a novel lncRNA that has been reported to play a vital role in cancers such as hepatocellular and renal cell carcinoma.^9^, ^10^ MicroRNAs (miRNAs) are a class of evolutionarily highly conserved ncRNA molecules that play vital roles in various physiological processes such as cell proliferation, cell cycle, and apoptosis.^11^ A previous study has found that hsa‐miR‐1207‐5p is potentially related to OA.^12^ LncRNAs regulate target genes by acting on miRNAs, thereby affecting the transcription of target genes.^13^ The lncRNA/miRNA axis has been widely studied in the progression of various human diseases, including OA.^14^, ^15^ However, the role of LINC01138/hsa‐miR‐1207‐5p in OA has not yet been studied.

The JAK/STAT3 signaling pathway can be triggered by pro‐inflammatory cytokines and is involved in several physiological changes, such as inflammation, oxidative stress, cell growth, and apoptosis.^16^ Activation of the JAK/STAT3 signaling pathway can cause chondrocyte destruction, subchondral bone sclerosis, synovial cell proliferation, and synovitis, leading to the occurrence and development of OA.^17^, ^18^, ^19^ As a powerful regulatory mechanism for adult bone development and homeostasis, the Wnt signaling pathway is important in regulating cartilage cells and maintaining the health and integrity of the cartilage matrix.^20^ In the present study, the effects of LINC01138 and its mechanism of action in a cell model of OA were investigated. The results found here provide a theoretical basis for further development of targeted drugs for the treatment of OA.

## 2 METHODS

### 2.1 Specimen selection

Articular cartilage tissue specimens were collected from 45 patients (aged 54–71 years) who were admitted to the hospital for artificial joint replacement owing to severe joint OA. Cartilage tissues from the normal control group were collected from 28 patients (aged 24–59 years) without OA who had been amputated owing to acute trauma. This study was approved by the Ethics Committee of the Cancer Institute of our hospital. The inclusion criteria were as follows: patients who received a diagnosis of Kellgren/Lawrence radiographic grade III to IV OA, and obtainment of written informed consent from each patient. The exclusion criteria were as follows: patients with rheumatoid arthritis or other inflammatory arthritis and patients who received drug treatment that may influence cartilage metabolism, such as, corticosteroids. Regions of all articular surfaces of femur tissues discarded during artificial joint replacement and amputation were partitioned with a #10 blade and graded clinically by surgeons according to the Outerbridge classification.^21^, ^22^ A small portion of the pooled grades of cartilage from each subject was randomly selected and used for subsequent studies.

### 2.2 Chondrocyte culture

The collected human cartilage was cut into pieces of approximately 2 mm^2^. Following three washes with PBS, 0.25% trypsin (Sigma‐Aldrich, St. Louis) was added for incubation at 37°C for 1 h. The tissues were digested with 0.02% type II collagenase (Sigma‐Aldrich) at 37°C for 20 h. The digestion solution was passed twice through a 70 μm filter and centrifuged at 1200*g* for 5 min. The collected cells were seeded into 75 cm^2^ flasks and cultured in dulbecco's modified eagle medium (DMEM; Gibco) supplemented with 10% fetal bovine serum (Gibco) and 0.1% penicillin and streptomycin solution. The culture medium was renewed every 2–3 days. The expanded cells at passage 4–7 were used for the following experiments.

### 2.3 Bioinformatics analysis

The GSE1919 and GSE105027 datasets were selected for targeted chip research from the Gene Expression Omnibus (GEO) database (http://www.ncbi.nlm.nih.gov/geo/). Differentially expressed genes were screened using the “limma” package. The target genes of LINC01138 and hsa‐miR‐1207‐5p were searched for using DIANA‐TarBase (version 7.0; http://diana.imis.athenainnovation.gr/DianaTools) and TargetScan (version 7.1; http://www.targetscan.org/vert_71), respectively. To comprehensively analyze the basic functions and participating pathways mediating KIAA0101‐related OA, gene ontology (GO) term function annotation and Kyoto Encyclopedia of Genes and Genomes (KEGG) pathway enrichment analyses were performed using the ClusterProfiler package in R language.

### 2.4 Cell transfection

The LINC01138 specific short hairpin RNAs (shRNAs) and control shRNAs were synthesized and produced by GenePharma. The recombinant lentiviral expression vector, pCDH‐LINC01138‐EGFP, was constructed using an accurate PCR‐based synthesis method. hsa‐miR‐1207‐5p mimics, hsa‐miR‐1207‐5p inhibitor, and hsa‐miR‐1207‐5p inhibitor‐negative control (inhibitor‐NC) were purchased from GenePharma Co., Ltd. Small interfering RNAs (siRNAs) specifically targeting KIAA010 and scrambled negative control siRNA (si‐NC) were synthesized and purified using RiboBio. Transfection was performed using Lipofectamine 2000 (Invitrogen) according to the manufacturer's instructions.

### 2.5 Luciferase reporter assays

The sequences of wild‐type or mutant LINC01138 (KIAA010) 3'‐UTR including hsa‐miR‐1207‐5p binding sites were sub‐cloned into the pMIR‐luciferase reporter construct (Invitrogen) and each construct was cotransfected with hsa‐miR‐1207‐5p mimic or hsa‐miR‐1207‐5p‐NC into chondrocytes using Lipofectamine 2000 (Invitrogen). After culturing for 48 h, the cells were evaluated using a Dual‐Luciferase Reporter Assay System (Yesen). The luminescence values of the cells in each group were determined using Renilla luminescence activity as an internal reference.

### 2.6 Sulphated glycosaminoglycans (sGAG) analysis

The supernatant of the cell culture medium was collected and centrifuged at 800*g* for 5 min. The supernatant was mixed with 500 μl buffer solution and digested at 55°C for 8 h. A dimethyl blue solution (2.5 ml) was added to 200 μl supernatant for color development. The absorbance value was measured at 525 nm, and the content glycolaminesulfate polysaccharide sGAG in the cell culture medium was calculated.

### 2.7 Enzyme‐linked immunosorbent assay (ELISA)

The levels of matrix metalloproteinase (MMP)‐13, interleukin (IL)‐6 and tumor necrosis factor (TNF)‐α in the cell supernatants were measured using a human matrix metallopeptidase 13 ELISA kit (ZN2333; Baiao), human IL‐6 ELISA kit (E‐EL‐H0102c; elabscience) and human TNF‐α ELISA kit (TBK5020000601; TB Healthcare), respectively.

### 2.8 RNA extraction and quantitative RT‐PCR

Total RNA was isolated from OA tissues or human chondrocytes by homogenization using TRIzol reagent (Invitrogen). cDNA synthesis was performed using a Reverse Transcription Kit (Beijing TransGen Biotech Co., Ltd.). The expression levels were detected using SYBR green (SinoBio). Fold changes in gene expression are represented using 2^−ΔΔCt^. The primers sequences used are listed as follows: LINC01138, forward, 5'‐ACATCGTGAGCACATTTGAGA‐3', reverse: 5'‐TCTTG CTGTTCAGGGTGGTA‐3'; miR‐1207‐5p, forward, 5'‐GCGGCGGTGGC AGGGAGGCTGG‐3', reverse: 5'‐ATCCAGTGCAGGGTCCGAGG‐3'; KIAA0101, forward, 5'‐TCCTGAAGAGGCAGGAAGCAGT‐3', reverse: 5'‐TTGTGTGATC AGGTTGCAAAGGA‐3'; Col2α1, forward, 5'‐CTGGCTCCCAACACTGCCAA CGTC‐3', reverse: 5'‐TCCTTTGGGTTTGCAACGATTGT‐3'; aggrecan, forward, 5'‐TGAGGAGGGCTGGAACAAGTACC‐3', reverse: 5'‐GGAGGTGGTAATTGCA GGGAACA‐3'; GAPDH (internal control for mRNAs) forward, 5'‐CATGT TGCAACCGGGAAGGA‐3', reverse, 5'‐GCCCAATACGACCAAATCAGAG‐3'; U6 (internal control for miRNA) forward, 5'‐CTCGCTTCGGCAGCACA‐3', reverse, 5'‐AACGCTTCACGAATTTGCGT‐3'.

### 2.9 Western blot analysis

Proteins were extracted using RIPA Lysis Solution (P0013C; Beyotime), and the protein concentration was measured using the BCA protein kit (KeyGen Biotech Co., Ltd). The proteins were separated by using SDS‐PAGE and transferred onto polyvinylidene fluoride membranes. The membrane was then incubated with the following primary antibodies: MMP‐13 (1:1000; 18165‐1‐AP, Proteintech), IL‐6 (1:500; 21865‐1‐AP, Proteintech), TNF‐α (1:500; 17590‐1‐AP, Proteintech), JAK1 (1:1000; ab133666, Abcam), p‐JAK1 (1:1000; ab138005, Abcam), STAT3 (1:1000; ab68153, Abcam), p‐STAT3 (1:1000; #9131; Cell Signaling Technology), Wnt1 (1:1000; ab15251, Abcam), β‐catenin (1:500; ab223075, Abcam), and GAPDH (1:1000; ab9485, Abcam). The next day, the membranes were incubated with the secondary antibody, and the intensity of protein expression was detected using ECL chemiluminescence (Beyotime).

### 2.10 Statistical analysis

The data were analyzed using SPSS 19.0 (SPSS Inc.). Unpaired Student's *t*‐test and one‐way analysis if variance were used to assess differences between groups. Statistical significance was set at *p* < .05.

## 3 RESULTS

### 3.1 LINC01138 was highly expressed in OA tissues

**Figure 1:**
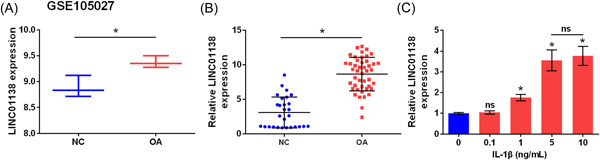
LINC01138 was highly expressed in OA tissues. (A) LINC01138 level in GSE105027 data set was analyzed in GEO database. (B) Expression level of LINC01138 in OA patient tissue was determined by qRT‐PCR. (C) Expression level of LINC01138 in chondrocytes treated with IL‐1β was detected by qRT‐PCR. *p < .05 compare with normal (NC) group. ns, no significant; qRT‐PCR, Quantitative real time PCR.

GEO data analysis indicated that LINC01138 expression was significantly higher in the tissues of patients with OA than in the NC group (*p* < .05; Figure 1A). Meanwhile, quantitative real time PCR (qRT‐PCR) analysis suggested that LINC01138 was highly expressed in the tissues of patients with OA compared to that in the NC group (*p* < .05; Figure 1B). Furthermore, qRT‐PCR results showed that different concentrations of IL‐1β led to an increase in LINC01138 expression in primary chondrocytes (*p* < .05; Figure 1C). Specifically, 5 ng/ml of IL‐1β was selected as the optimal concentration and used in subsequent experiments. Taken together, LINC01138 was overexpressed in patients with OA, and IL‐1β increased the expression of LINC01138 in human chondrocytes.

### 3.2 Silencing of LINC01138 protected chondrocytes from ECM degradation and inflammation caused by IL‐1β

**Figure 2:**
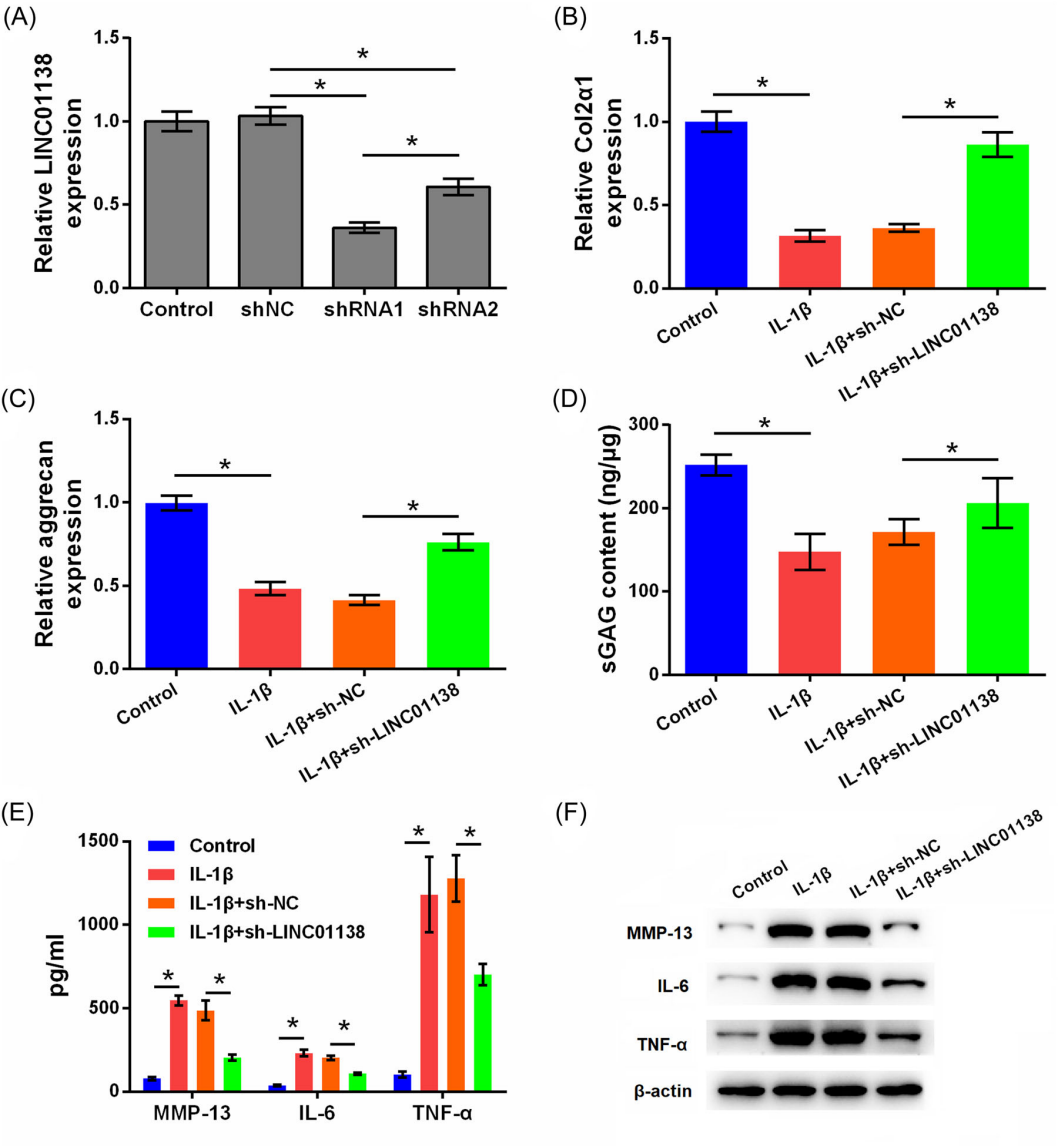
Silencing of LINC01138 protected chondrocytes from matrix degradation and inflammation caused by IL‐1β. (A) Expression level of LINC01138 in cells was determined by qRT‐PCR. (B) Level of Col2α1 and (C) aggrecan in cells was determined by qRT‐PCR. (D) Level of sGAG in cells was determined by sGAG analysis. (E) Levels of MMP‐13, IL‐6, and TNF‐α in cell supernatant were determined by ELISA. (F) Protein levels of MMP‐13, IL‐6, and TNF‐α in cells were determined by western blot. *p < .05 as compared with the indicated group. ELISA, enzyme‐linked immunosorbent assay; IL, interleukin; MMP, matrix metalloproteinase; qRT‐PCR, Quantitative real time PCR; sGAG, sulphated glycosaminoglycans; TNF, tumor necrosis factor.

To silence LINC01138, cells were transfected with two shRNAs (1, 2) of different sequences into chondrocytes; qRT‐PCR analysis showed that the level of LINC01138 was markedly downregulated in the shRNA1 and shRNA2 groups, and LINC01138 levels in the shRNA1 group were lower than those in the shRNA2 group (*p* < .05; Figure 2A). Considering that the transfection efficiency of shRNA1 was higher, shRNA1 was selected for use in subsequent experiments. Compared to the control group, IL‐1β significantly inhibited the levels of Col2α1, aggrecan, and sGAG (*p* < .05). However, the levels of Col2α1, aggrecan, and sGAG were increased in the IL‐1β + sh‐LINC01138 group compared to those in the IL‐1β + sh‐NC group (*p* < .05; Figure 2B–D). In addition, IL‐1β significantly increased the secretion of MMP‐13, IL‐6, and TNF‐α (*p* < .05). Compared with the IL‐1β + sh‐NC group, the secretion levels of MMP‐13, IL‐6, and TNF‐α were significantly inhibited in the IL‐1β + sh‐LINC01138 group (*p* < .05; Figure 2E). Furthermore, IL‐1β increased the protein levels of MMP‐13, IL‐6, and TNF‐α, and LINC01138 knockdown exerted the opposite effect (*p* < .05; Figure 2F). Collectively, silencing LINC01138 protected chondrocytes from ECM degradation and inflammation induced by IL‐1β.

### 3.3 LINC01138 targeted hsa‐miR‐1207‐5p

**Figure 3:**
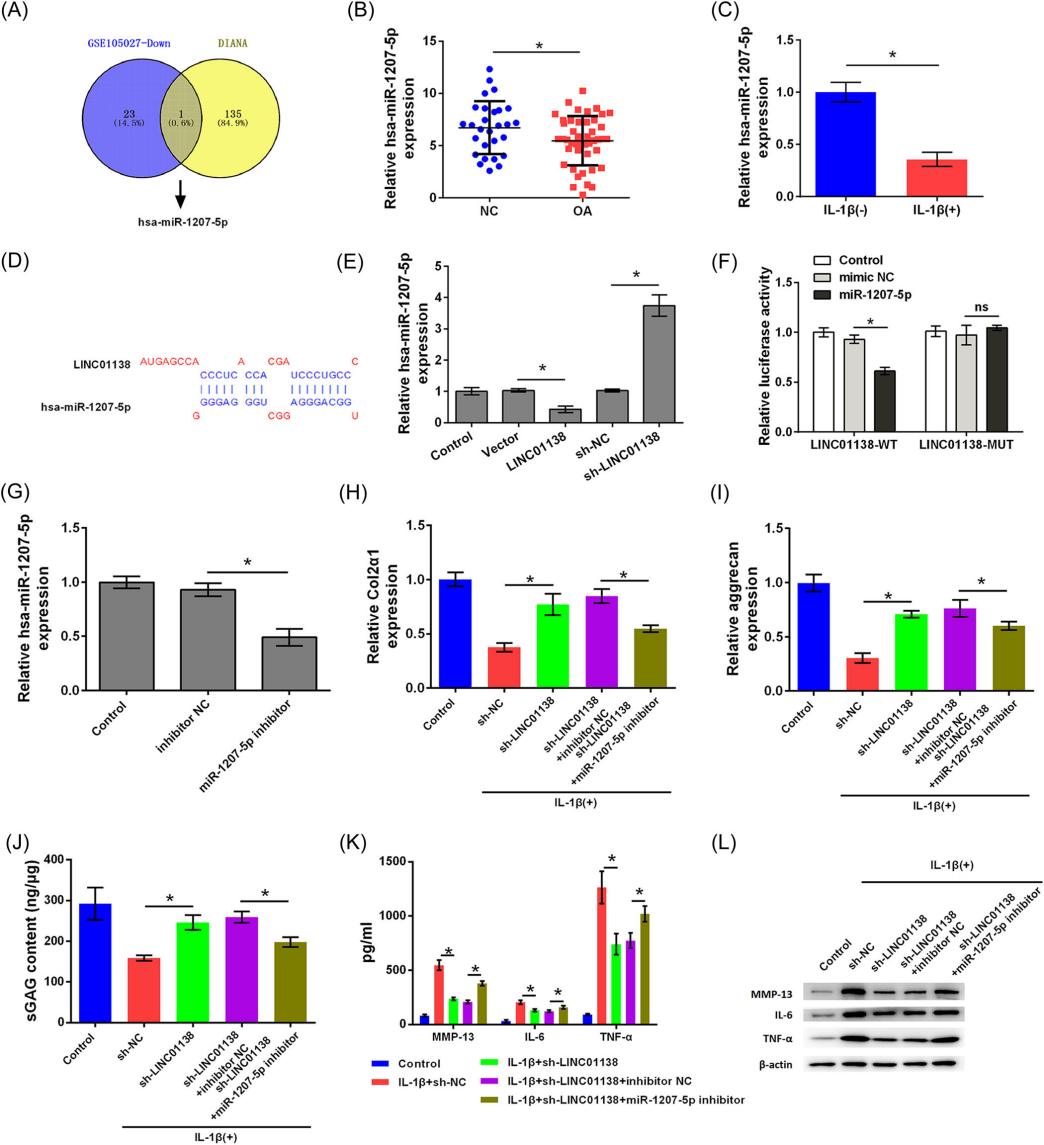
LINC01138 targeted hsa‐miR‐1207‐5p. (A) The differentially expressed miRNAs in GSE105027 data set was analyzed and target genes of the LINC01138 were searched using the DIANA‐TarBase. hsa‐miR‐1207‐5p was screened out as an overlapped gene. (B) Expression level of hsa‐miR‐1207‐5p in OA patient tissue was determined by qRT‐PCR. (C) Expression level of hsa‐miR‐1207‐5p in chondrocytes treated with IL‐1β was detected by qRT‐PCR. (D) Binding sites between LINC01138 and miR‐1207‐5p were predicted by DIANA‐TarBase. (E) Expression level of miR‐1207‐5p was detected by qRT‐PCR. (F) Luciferase activity was detected by Dual‐luciferase reporter assay. (G) Transfection efficient was determined by qRT‐PCR. (H) Levels of Col2α1 and (I) aggrecan in cells were determined by qRT‐PCR. (J) Level of sGAG in cells was determined by sGAG analysis. (K) The levels of MMP‐13, IL‐6, and TNF‐α in cells were determined by ELISA. (L) Protein levels of MMP‐13, IL‐6, and TNF‐α in cells were determined by western blot analysis. ^*^ *p* < .05 as compared with the indicated group. ELISA, enzyme‐linked immunosorbent assay; IL, interleukin; miRNA, microRNA; qRT‐PCR, Quantitative real time PCR; sGAG, sulphated glycosaminoglycans; TNF, tumor necrosis factor.

We searched for miRNA expression in OA in the GSE105027 data set of the GEO database and predicted the target miRNA molecules that have LINC01138‐binding site through the DIANA‐TarBase database. Hsa‐miR‐1207‐5p was expressed at low levels in OA and hsa‐miR‐1207‐5p had a complementary binding site with LINC01138 (Figure 3A). qRT‐PCR analysis suggested that miR‐1207‐5p was expressed at lower levels in tissues of patients with OA than in the NC group (*p* < .05; Figure 3B). In addition, miR‐1207‐5p expression was markedly inhibited in primary chondrocytes treated with IL‐1β (*p* < .05; Figure 3C). Figure 3D shows the target binding sites between LINC01138 and miR‐1207‐5p which were analyzed using DIANA‐TarBase prediction software. Moreover, overexpression of LINC01138 inhibited the expression of hsa‐miR‐1207‐5p, whereas silencing LINC01138 increased hsa‐miR‐1207‐5p expression (*p* < .05; Figure 3E). The results of the luciferase reporter assay indicated that hsa‐miR‐1207‐5p may be a downstream target of LINC01138 (*p* < .05; Figure 3F). Taken together, LINC01138 targets hsa‐miR‐1207‐5p.

Subsequent studies investigated whether LINC01138 silencing protects chondrocytes from ECM degradation and inflammation caused by IL‐1β by regulating hsa‐miR‐1207‐5p. As shown in Figure 3G, hsa‐miR‐1207‐5p expression levels were inhibited in cells transfected with hsa‐miR‐1207‐5p inhibitor, indicating that cells transfected with hsa‐miR‐1207‐5p inhibitor successfully (*p* < .05). In addition, the levels of Col2α1, aggrecan, and sGAG were higher in the IL‐1β + sh‐LINC01138 group than in the IL‐1β + sh‐NC group (*p* < .05). Compared with the IL‐1β + sh‐LINC01138‐inhibitor NC group, the levels of Col2α1, aggrecan, and sGAG in the IL‐1β + sh‐LINC01138 + miR‐1207‐5p inhibitor group were markedly decreased, indicating that silencing hsa‐miR‐1207‐5p alleviated the effect of LINC01138 silencing on Col2α1, aggrecan, and sGAG expression (*p* < .05; Figure 3H‐J). Furthermore, the production and release of MMP‐13, IL‐6, and TNF‐α in the IL‐1β + sh‐LINC01138 group were lower than those in the IL‐1β + sh‐NC group (*p* < .05). However, compared with that in the IL‐1β + sh‐LINC01138 + inhibitor NC group, the production and release of MMP‐13, IL‐6, and TNF‐α in the IL‐1β + sh‐LINC01138 + miR‐1207‐5p inhibitor group increased significantly, suggesting that knockdown of hsa‐miR‐1207‐5p reversed the effect of LINC01138 silencing on MMP‐13, IL‐6, and TNF‐α levels (*p* < .05; Figure 3K,L). Collectively, silencing of LINC01138 protected chondrocytes from ECM degradation and inflammation caused by IL‐1β by regulating hsa‐miR‐1207‐5p.

### 3.4 Hsa‐miR‐1207‐5p targeted KIAA0101

**Figure 4:**
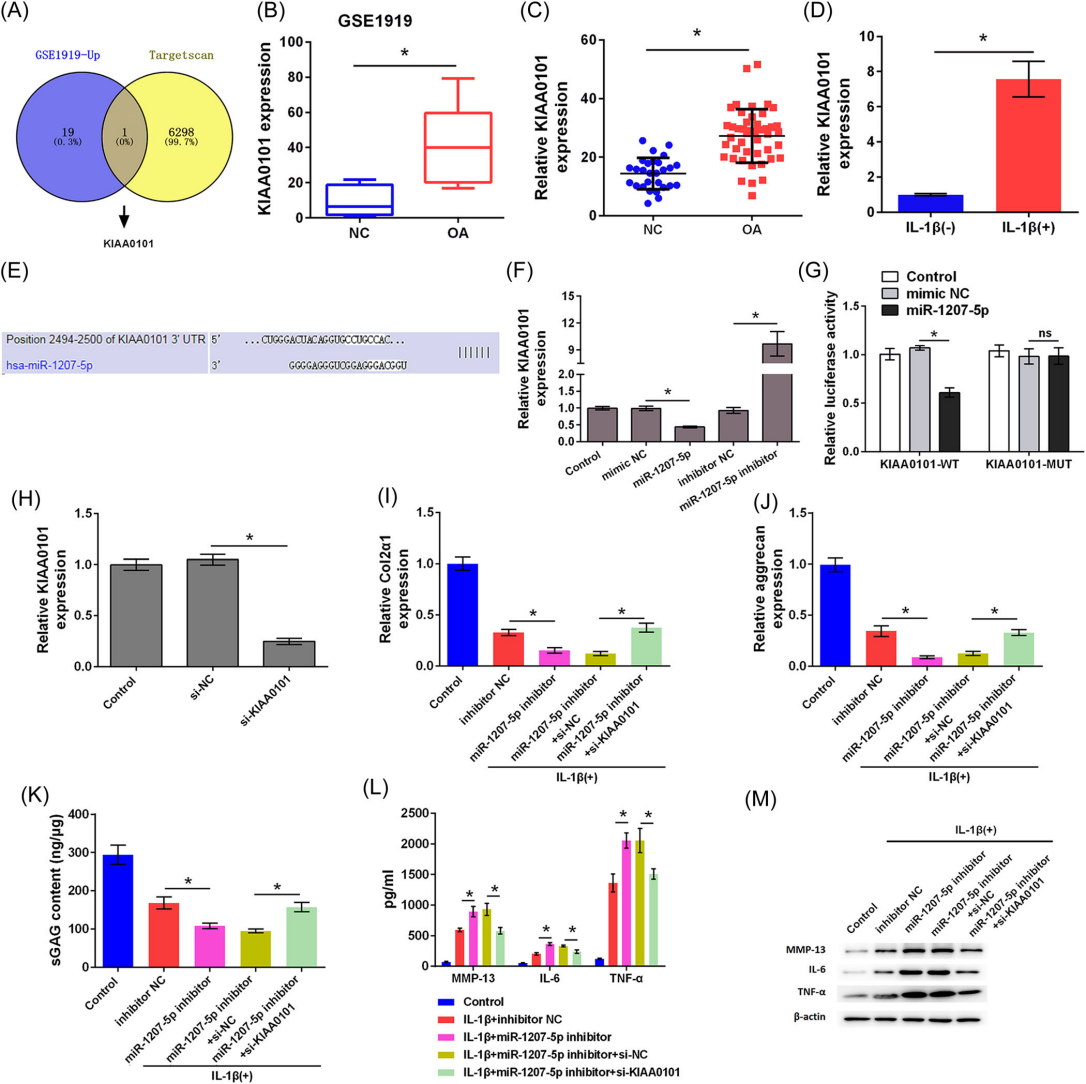
Hsa‐miR‐1207‐5p targeted KIAA0101. (A) Differential expressed genes in GSE1919 data set were searched and target genes of hsa‐miR‐1207‐5p were searched using Targetscan. KIAA0101 was screened out as an overlapped gene. (B) KIAA0101 level in GSE1919 data set was analyzed. (C) Expression level of KIAA0101 in OA patient tissues was determined by qRT‐PCR. (D) Expression level of KIAA0101 in chondrocytes treated with IL‐1β was detected by qRT‐PCR. (E) Binding sites between KIAA0101 and miR‐1207‐5p were predicted by Targetscan. (F) Expression level of KIAA0101 was detected by qRT‐PCR. (G) Luciferase activity was detected by Dual‐luciferase reporter assay. (H) Transfection efficient was determined by qRT‐PCR. (I) Expression levels of Col2α1 and (J) aggrecan in cells were determined by qRT‐PCR. (K) Level of sGAG in cells was determined by sGAG analysis. (L) The levels MMP‐13, IL‐6, and TNF‐α in cells were determined by ELISA. (M) Protein levels of MMP‐13, IL‐6, and TNF‐α in cells were determined by western blot analysis. ^*^ *p* < .05 as compared with the indicated group. ELISA, enzyme‐linked immunosorbent assay; IL, interleukin; qRT‐PCR, Quantitative real time PCR; sGAG, sulphated glycosaminoglycans; TNF, tumor necrosis factor.

The expression of mRNA in OA was researched in the GSE1919 data from the GEO database, and target mRNA molecules that can bind with hsa‐miR‐1207‐5p were predicted using the TargetScan database. KIAA0101 was highly expressed in OA and had a complementary binding site with hsa‐miR‐1207‐5p, suggesting that hsa‐miR‐1207‐5p may exert its function through KIAA0101 (Figure 4A). GEO analysis showed that KIAA0101 was significantly highly expressed in patients with OA (*p* < .05; Figure 4B). KIAA0101 was highly expressed in OA tissues compared to that in the NC group (*p* < .05; Figure 4C). KIAA0101 was highly expressed in primary chondrocytes treated with IL‐1β (*p* < .05; Figure 4D). Furthermore, the target complementary pairing sequences of hsa‐miR‐1207‐5p and KIAA0101 were analyzed using the TargetScan prediction software (Figure 4E). Overexpression of hsa‐miR‐1207‐5p caused low expression of KIAA0101 in chondrocytes, whereassilencing hsa‐miR‐1207‐5p caused high expression of KIAA0101 (*p* < .05; Figure 4F). Luciferase reporter assay results verified that hsa‐miR‐1207‐5p could bind to KIAA0101 (*p* < .05; Figure 4G). These data indicate that hsa‐miR‐1207‐5p targeted KIAA0101.

Furthermore, we investigated whether hsa‐miR‐1207‐5p silencing accelerated the damage caused by IL‐1β to chondrocytes by regulating KIAA0101. As shown in Figure 4H, KIAA0101 expression level was decreased in the si‐KIAA0101 group compared to that in the si‐NC group, indicating successful transfection (*p* < .05). Compared to the IL‐1β + inhibitor NC group, Col2α1, aggrecan, and sGAG levels were markedly inhibited in the IL‐1β + miR‐1207‐5p inhibitor group (*p* < .05). However, Col2α1, aggrecan, and sGAG levels were increased in the IL‐1β + miR‐1207‐5p inhibitor+si‐KIAA0101 group compared to those in the IL‐1β + miR‐1207‐5p inhibitor+si‐NC group, suggesting that silencing KIAA0101 reversed the effect of silencing hsa‐miR‐1207‐5p on Col2α1, aggrecan, and sGAG expression (*p* < .05; Figure 4I‐K). Furthermore, compared with the IL‐1β + inhibitor NC group, the levels of MMP‐13, IL‐6, and TNF‐α were increased in the IL‐1β + miR‐1207‐5p inhibitor group (*p* < .05). However, the levels of MMP‐13, IL‐6, and TNF‐α in the IL‐1β + miR‐1207‐5p inhibitor+si‐KIAA0101 group were lower than those in the IL‐1β + miR‐1207‐5p inhibitor+si‐NC group, indicating that silencing KIAA0101 alleviated the effect of silencing hsa‐miR‐1207‐5p on the levels of MMP‐13, IL‐6, and TNF‐α (*p* < .05; Figure 4L,M). Taken together, silencing hsa‐miR‐1207‐5p accelerated the damage caused by IL‐1β to chondrocytes by regulating KIAA0101.

### 3.5 Silencing KIAA0101 inhibited the JAK/STAT and Wnt signaling pathways

**Figure 5:**
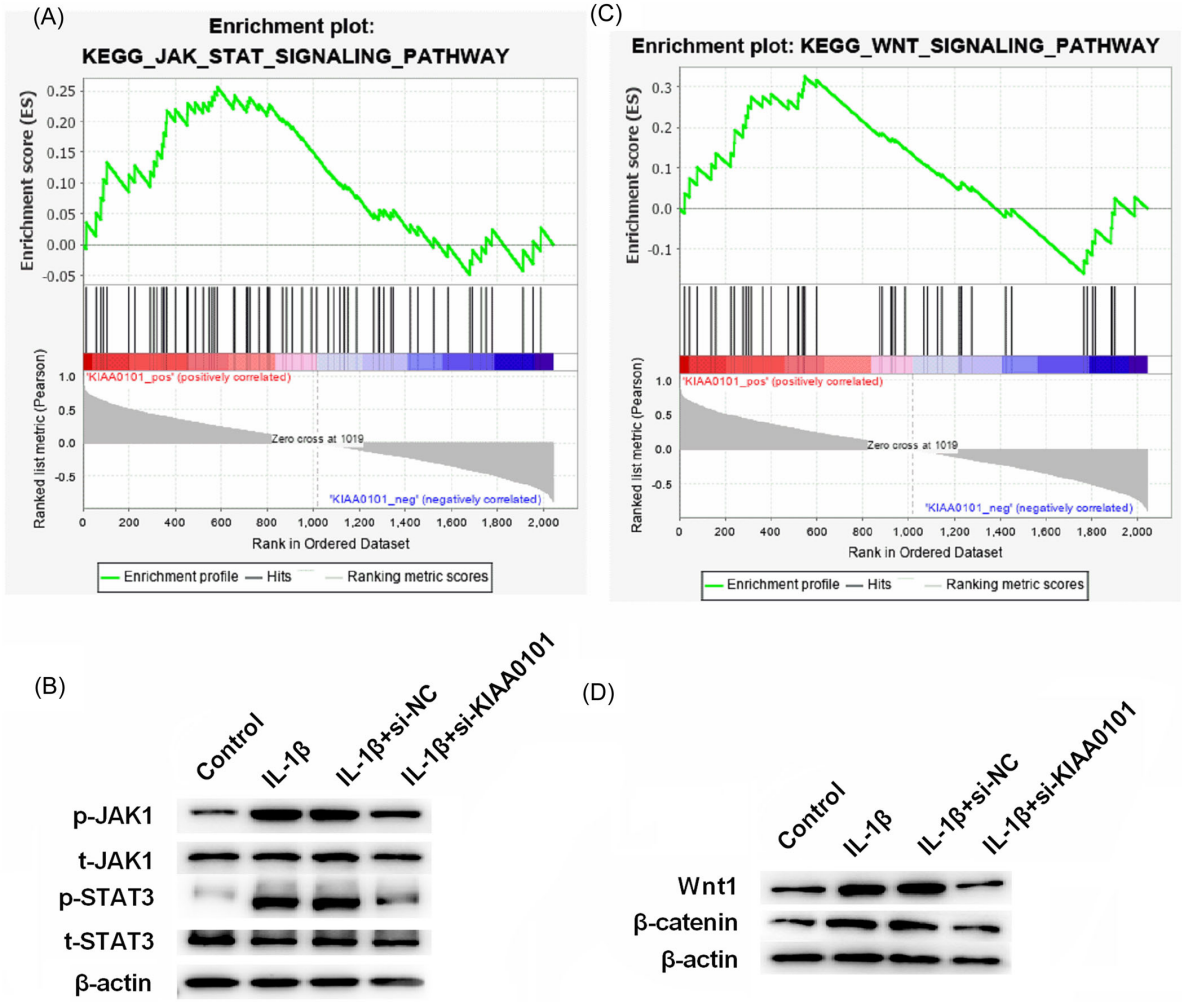
Silencing of KIAA0101 inhibits the JAK/STAT and Wnt signaling pathways. (A, C) Pathways activated by KIAA0101 were obtained by GSEA analysis. (B, D) Protein levels of JAK1, p‐JAK1, STAT3, p‐STAT3, Wnt1, and β‐catenin were measured by western blotting.

GSEA software was used to analyze the expression data of chip GSE1919, and the pathways activated by KIAA0101 were identified. Considering that JAK/STAT and Wnt are widely recognized as two important pathways involved in cellular inflammation, these two pathways were selected for further research (Figures 5A,C). Western blot analysis results showed that, silencing of KIAA0101 inhibited the phosphorylation of JAK1 and STAT3, as well as the protein expression of Wnt1 and β‐catenin (Figures 5B,D). Collectively, silencing KIAA0101 inhibited the JAK/STAT and Wnt signaling pathways.

## 4 DISCUSSION

IL‐1β has been considered the most important and widely used method for inducing chondrocyte apoptosis, inflammation, and ECM degeneration in OA experimental models.^23^ In the present study, primary human chondrocytes were treated with IL‐1β to establish an OA cell model. LINC01138 is highly expressed in OA tissues and in an OA cell model. LINC01138 silencing effectively inhibited ECM degeneration and chondrocyte inflammation. LINC01138 plays a role in the progression of OA, possibly by regulating the hsa‐miR‐1207‐5p/KIAA0101 axis.

ECM degradation is the main feature of OA, and the main components of ECM include Col2 and proteoglycan.^24^ During OA, the synthesis of aggrecan, Col2α1, and sGAG decreases, while the synthesis of MMP‐13 increases.^25^ In the current study, in response to IL‐1β, the levels of aggrecan, Col2α1, and sGAG in chondrocytes decreased, which was consistent with a previous study.^26^ Previous studies have shown that the expression of MMP‐13 and related inflammatory cytokines (IL‐6 and TNF‐α) in OA was upregulated, and the expression was continuously upregulated as the degree of deterioration increased.^27^, ^28^ The downregulation of LINC01138 protected chondrocytes from IL‐1β‐induced damage by regulating aggrecan, Col2α1, sGAG, and cytokines, indicating that silencing LINC01138 improved the development of OA.

It has been reported that lncRNAs exert their biological functions by acting as natural miRNA sponges.^29^, ^30^ For example, lncRNA SNHG7 bind to miR‐34a‐5p to regulate cell proliferation, apoptosis, and autophagy by targeting SYVN1.^30^ Downregulation of lncRNA SNHG16 remarkably inhibits the progression of OA by sponging miR‑373‑3p.^31^ In the present study, LINC01138 was found to be an upstream regulator of hsa‐miR‐1207‐5p. Silencing LINC01138 protected chondrocytes from IL‐1β‐induced ECM degradation and inflammation by regulating hsa‐miR‐1207‐5p. Furthermore, KIAA0101 was overexpressed in OA and found to be a downstream target of hsa‐miR‐1207‐5p. KIAA0101 is a nuclear protein that is involved in cell proliferation, differentiation, migration, apoptosis, metabolism, and repair after DNA damage.^32^ Overexpression of KIAA0101 in tumor eventually leads to tumor recurrence and death, which is an important factor for poor prognosis.^32^, ^33^ Previous reports have shown that KIAA0101 is a target gene of miRNAs.^34^, ^35^ In this study, we found that silencing of KIAA0101 eliminated the effect of hsa‐miR‐1207‐5p silencing on chondrocytes.

Several signaling pathways are involved in OA, including the MAPK, PI3K/Akt, Wnt/β‐catenin, Fas/FasL, NF‐κB, and JAK/STAT signaling pathways.^28^, ^36^, ^37^, ^38^, ^39^ In the present study, JAK/STAT and Wnt were found to be two downstream pathways of KIAA0101 in OA. KIAA0101 was reported to improve the migration and chemotherapy resistance of epithelial ovarian cancer cells by regulating Wnt/β‐catenin signaling.^34^ The Wnt signaling pathway is inhibited in normal chondrocytes, and once activated, causes OA cartilage damage.^40^ miRNA‐320c inhibits OA development by downregulating the Wnt signaling pathway.^41^ Activation of the JAK/STAT signaling pathway leads to OA progression and imbalance in cartilage ECM metabolism.^42^ STAT3 is a potent transcription factor that regulates the expression of several target genes involved in the pathogenesis of OA, including proteases, cytokines, chemokines, and key regulators of chondrocyte proliferation, differentiation, and apoptosis. In the current study, silencing of KIAA0101 inhibited the JAK/STAT and Wnt signaling pathways, indicating that the downregulation of KIAA0101 eliminated the effect of silencing hsa‐miR‐1207‐5p on chondrocytes by inhibiting the JAK/STAT and Wnt signaling pathways.

The present study has several limitations. First, we included human cartilage samples and observed a significant difference in LINC01138 expression between OA tissues and normal controls. However, because healthy cartilage samples are generally difficult to obtain, the ages of donors with and without OA do not overlap significantly (54–71 years vs. 24–59 years). Considering that OA is an ageing‐associated disease, further studies are required to verify LINC01138 expression in age‐matched patients with OA and normal controls. Second, targeting of lncRNAs has been widely accepted to be achieved through blocking or knockdown by antisense oligonucleotides, siRNAs, or shRNAs.^43^ In the current study, LINC01138 expression was silenced by transfection with specific shRNAs. Similarly, silencing of LINC01138 using specific siRNAs have been widely reported.^10^, ^44^, ^45^ However, the prospective class of drugs and dosage forms that can shut down LINC01138 require further investigation.

## 5 CONCLUSION

LINC01138 was significantly highly expressed in the tissues of patients with OA, and silencing of LINC01138 protected chondrocytes from IL‐1β‐induced damage possibly by regulating the hsa‐miR‐1207‐5p/KIAA0101 axis. The findings of the present study provide a theoretical basis for further development of targeted drugs for the treatment of OA.

## AUTHOR CONTRIBUTIONS

**Jiangtao Zhang**: Conception and design; data analysis and interpretation; manuscript writing; final approval of manuscript. **Genbing Lv**: Perform research; manuscript writing; final approval of manuscript.

## CONFLICT OF INTEREST

The authors declare no conflict of interest.

## ETHICS STATEMENT

The protocol of this research has been approved by the Ethics Committee of Sun Si Miao Hospital of Beijing University of Chinese Medicine, Tongchuan Traditional Chinese Medicine Hospital (SX2020T11‐04). All patients have signed written informed consent.

## Data Availability

The datasets used and analyzed during the current study are available from the corresponding author on reasonable request.
